# Hemophagocytic Lymphohistiocytosis Syndrome With Hepatic Involvement and Secondary to Acute B-cell Lymphocytic Leukemia: A Case Report

**DOI:** 10.7759/cureus.28620

**Published:** 2022-08-31

**Authors:** Bushra A Bangash, Farrah Alarmanazi, Arseniy Atlanov, Christian C Toquica Gahona, Banu Farabi

**Affiliations:** 1 Internal Medicine, St. Peter’s University Hospital, New Brunswick, USA; 2 Dermatology, New York Medical College, Metropolitan Hospital Center, New York, USA

**Keywords:** hematologic malignancies (hm), secondary hlh, acute fulminant liver failure, hyperinflammatory response, macrophage activation syndrome (mas)

## Abstract

Hemophagocytic lymphohistiocytosis (HLH) is a hyperactivation syndrome associated with the overactivation of macrophages, which produce enormous amounts of tumor necrosis factor-alpha and interferon-gamma. HLH often presents with diminished T-cell and natural killer (NK) cell regulation, which can develop due to underlying genetic causes, infections, autoimmune diseases, and/or secondary to malignancies. Here, we describe the case of a 39-year-old man who presented with subjective fevers and fatigue. Further workup revealed hyperferritinemia, hypertriglyceridemia, and absent NK-cell activity, which raised a strong suspicion for HLH. The workup also revealed elevated aminotransferases signaling hepatic involvement that was attributed to HLH. Bone marrow biopsy revealed hypercellularity instead of the hemophagocytosis usually seen in HLH. Flow cytometry revealed acute B-cell lymphocytic leukemia, which was identified as the cause of HLH in our patient. This case highlights the rare presentation of HLH secondary to a B-cell malignancy. It addresses the importance of high clinical suspicion in patients with high fevers despite the use of broad-spectrum antibiotics. There is limited information on the treatment of HLH secondary to malignancies specifically, and further research in this area is needed to increase the survival rate.

## Introduction

Hemophagocytic lymphohistiocytosis (HLH) is a syndrome of immune system hyperactivity leading to pathological inflammation. Defective macrophages, natural killer (NK) cells, and/or cytotoxic T-lymphocytes can be considered as the inciting factors for pathogenesis. In all varieties of HLH, macrophages are overactivated, releasing massive amounts of cytokines, which leads to organ and tissue damage [1]. A high degree of clinical suspicion is required to diagnose HLH due to the rarity and nonspecific presentation of the disorder. The diagnosis of HLH is based on the criteria used in the HLH-2004 trial. However, rarely do patients with HLH meet the strict criteria outlined in this trial. HLH most likely develops in adults secondary to malignancy, infections, or rheumatologic disorders [2]. Among these cases, hematologic malignancies are the most prevalent [3]. HLH in adults is most commonly associated with T-cell and NK cell lineage malignancies and is rarely reported secondary to B-cell malignancies [2]. In this paper, we describe a case of HLH with hepatic involvement arising in a 39-year-old male with no medical history or prior hospitalizations who presented with subjective fevers and fatigue. Our patient was found to have acute B-cell lymphocytic leukemia after a bone marrow biopsy. Further investigations revealed that the source of his unremitting fever was secondary to HLH.

## Case presentation

A 39-year-old male with no prior medical history or hospitalizations presented to the emergency department with a two-day history of subjective fevers and fatigue. The patient reported that he had been feeling hot despite moving items in and out of the freezer (10-20°F) at work. He never measured his temperature at home, but taking Motrin (Ibuprofen) did not alleviate his symptoms. Associated symptoms included an intermittent, mild, dry cough and palpitations. The patient denied any trauma, headache, vision changes, dizziness, chest pain, shortness of breath, abdominal pain, nausea, vomiting, bowel or urinary symptoms, rashes, or wounds. He also did not have any sick contacts, a recent history of travel, or changes in diet and health apart from the symptoms stated above. His vital signs on admission showed a temperature of 102.8°F, a pulse rate of 128 beats/minute, a respiratory rate of 31 breaths/minute, oxygen saturation of 99% on room air, and blood pressure of 118/68 mmHg. The physical examination was remarkable for scleral icterus, with a normal cardiopulmonary examination and no hepatosplenomegaly. In the emergency department, the patient was given 3 L of intravenous fluids and was started on empiric antibiotic therapy with ceftriaxone and azithromycin. Initial investigations revealed a white blood cell count of 2.3 × 10^3^/μL, red blood cell count 3.8 × 10^6^/μL, platelets 148 × 10^3^/μL, aspartate transaminase (AST) 1,163 U/L, alanine transaminase (ALT) 1,870 U/L, total bilirubin 3.9 mg/dL, lactic acid 2.1 mmol/L, ferritin >7,500 ng/mL, fibrinogen 583 mg/dL, lactate dehydrogenase 1,061 U/L, and triglycerides of 290 mg/dL (Table 1). An extensive infectious disease workup including human immunodeficiency virus, hepatitis B virus, hepatitis C virus, Lyme, cytomegalovirus, and Epstein-Barr virus was negative. Urinalysis and blood cultures were also negative. Computed tomography of the chest, abdomen, and pelvis was unremarkable. Ceftriaxone/azithromycin was later changed to doxycycline, piperacillin/tazobactam, acyclovir, and fluconazole, according to infectious disease recommendations given absolute neutropenia. Despite these interventions, the patient had high fevers of 102°F every day. On day three of the hospital stay, hematology was consulted, and the decision to perform a bone marrow biopsy was made. The biopsy revealed >95% blasts, a marked decrease in erythroid and myeloid precursors, and scant megakaryocytes. Flow cytometry showed 87% B-lymphoblasts, positive for CD10, CD19, CD20, CD22, CD45, CD34, HLA-DR, CD38, CD123, and CD71, and negative for CD117 and CD33. A final diagnosis of acute B-cell lymphocytic leukemia was made, and the patient was transferred to another hospital for induction of chemotherapy with subsequent bone marrow transplant without further complications.

**Table 1:** Laboratory investigations.

Test	Results	Reference values
White blood cell count	2.3 × 10^3^/μl	4–11 × 10^3^/µl
Red blood cell count	3.8 × 10^6^/μl	4.5–5.5 × 10^6^/μl
Hemoglobin	7.5 g/dL	11–15 g/dL
Absolute neutrophil count	400	2–7 × 10^3^/µL
Platelets	148 × 10^3^/μl	150–400 × 10^3^/µL
Aspartate transaminase	1,163 U/L	10–40 U/L
Alanine transaminase	1,870 U/L	10–50 U/L
Total bilirubin	3.9 mg/dL	0.1–1.2 mg/dL
Lactic acid	2.1 mmol/L	0.5–2.2 mmol/L
Ferritin	>7,500 ng/mL	12–300 ng/mL
Fibrinogen	583 mg/dL	200–400 mg/dL
Lactate dehydrogenase	1,061 U/L	<234 U/L
Triglycerides	290 mg/dL	<150 mg/dL

## Discussion

HLH is a hyperactivation syndrome with a severe uncontrolled inflammatory response. The underlying pathophysiology seems to be a diminished NK and T-cell function and an overactivation of macrophages which produce enormous amounts of tumor necrosis factor (TNF)-α and interferon-gamma (IFN)-γ. Bone marrow analysis may also show direct hemophagocytosis. The etiology is classified as either primary/familial or secondary/acquired. Primary HLH indicates an inherited version of the syndrome, where gene mutations lead to the development of dysfunctional cytotoxic lymphocytes; these patients mostly present in infancy. Secondary HLH indicates a reactive acquired disease that mainly presents in adults. It can develop due to loss of inhibitory immune mechanisms or due to a challenge to the immune system that leads to its dysfunction and overactivation. Examples of these antigen challenges include persistent infections, autoimmune diseases, or malignancy [4].

The disease is often challenging to diagnose because the symptoms are nonspecific and can be linked to many other diseases. The diagnosis of HLH is based on the criteria used in the HLH-2004 trial. Diagnosis of HLH can be established if there is molecular evidence (i.e., mutations in *PRF1*, *UNC13D*, *Munc18-2*, *Rab27a*, *STX11*, *SH2D1A*, or *BIRC4* genes), or if five of the eight criteria are met fever (≥38.5°C); splenomegaly; bicytopenias; hypertriglyceridemia (fasting levels >265 mg/dL) and/or hypofibrinogenemia (<150 mg/dL); hemophagocytosis in bone marrow, spleen, lymph nodes, or liver; low or absent NK-cell activity; ferritin >500 ng/mL; and elevated sCD25 (α-chain of IL-2 receptor) [5]. However, rarely do patients with HLH meet the strict criteria outlined in this trial, and, therefore, it is essential to maintain a high index of suspicion in patients presenting with any of the criteria listed above. Our patient had fevers above 38.5°C, cytopenias reflected by a hemoglobin level of 7.5 g/dL, and neutrophil count of 400, Triglyceride levels greater than 265 mg/dL, ferritin levels greater than 500 ng/mL, and peripheral blood flow cytometry showed absent NK-cell activity (CD56neg). Our patient fulfilled five of the eight criteria outlined, confirming the diagnosis of HLH (Table 2).

**Table 2:** Findings that satisfy HLH requirements. Our patient fulfilled 5 out of the 8 criteria for HLH. HLH: hemophagocytic lymphohistiocytosis; NK: natural killer

Findings in our patient	Reference range for HLH requirements
Fevers of 39.3°C	≥38.5°C
Bicytopenias	
Hemoglobin of 7.5 g/dL	<9 g/dL
Absolute neutrophil count of 400	<1,000/L
Hypertriglyceridemia	>265 mg/dL
Hyperferritinemia	>500 ng/mL
Absent NK cell activity	Decreased or absent NK cell activity

Our patient presented to the Emergency Department (ED) with vague symptoms such as fever and fatigue, which can yield an extensive list of differentials. Due to the high AST of 1,163 U/L and ALT of 1,870 U/L with a bilirubin of 3.9, viral hepatitis, autoimmune hepatitis, and systemic lupus erythematosus were also on our list of differentials. However, a viral hepatitis panel, anti-smooth muscle antibodies, and antinuclear antibodies were negative. Severe hepatic involvement with no specific cause, such as viral hepatitis or hepatotoxic drugs, could be attributed to HLH. A recent review of several case studies found that liver involvement was present in around 67% of those diagnosed with HLH [6].

Further infectious disease workup was negative, which ruled out the most common infectious causes for fever and fatigue. Adult Still disease was considered a possibility due to high fevers, hyperferritinemia, and abnormal liver function tests. However, this was less likely because the patient did not have arthralgias or the maculopapular evanescent salmon-pink skin rash [1].

**Figure 1:**
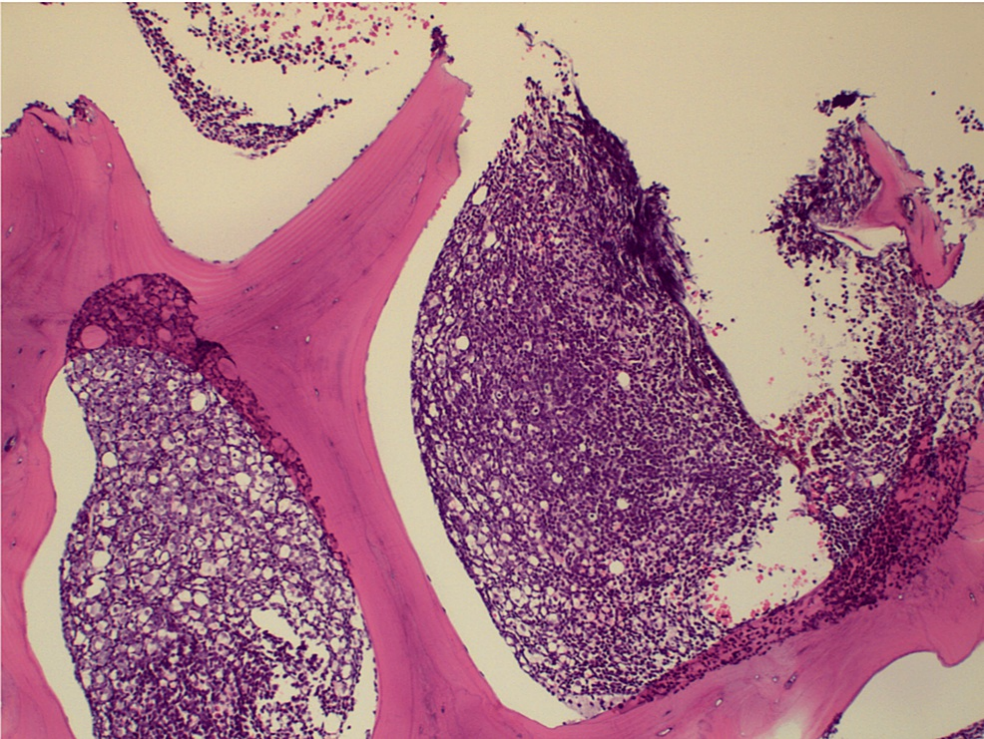
Bone marrow biopsy showing a hypercellular marrow (10×).

**Figure 2:**
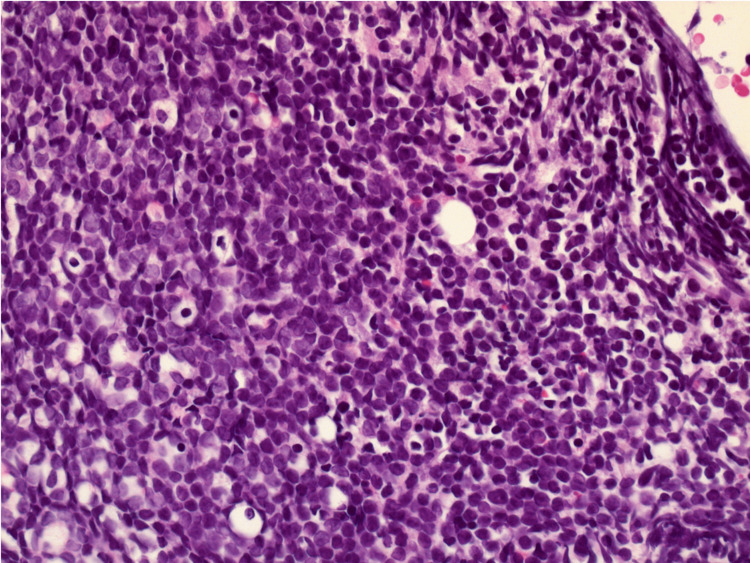
Hypercellular bone marrow secondary to acute B-cell lymphocytic leukemia (40×).

Due to the nonspecific symptoms of fever, fatigue, and cytopenias, we were suspicious of an underlying hematologic malignancy. A decision to perform a bone marrow biopsy was made after a hematologist-oncologist consultation. Bone marrow biopsy and flow cytometry confirmed a diagnosis of acute B-cell lymphocytic leukemia. In HLH, macrophage activation leads to the engulfment of hematopoietic cells, a process known as hemophagocytosis [3]. In our case, hemophagocytosis in bone marrow was absent, and a hypercellular marrow was seen due to acute B-cell lymphocytic leukemia (Figures 1, 2). The patient was transferred to another facility for induction chemotherapy with a subsequent bone marrow transplant.

Our patient’s HLH was most likely secondary to acute B-cell lymphocytic leukemia. Secondary HLH can occur in the setting of malignancy, infections, and autoimmune diseases. HLH is most commonly associated with hematologic malignancies associated with T cells and NK cells. Our case is unique because HLH has rarely been described in B-cell malignancies such as acute B-cell lymphocytic leukemia. HLH can occur at the onset or relapse of malignancy or during the chemotherapy phase due to immunosuppression. Our patient did not have a medical history of any malignancy; therefore, likely had HLH after the onset of acute B-cell lymphocytic leukemia. Furthermore, our case highlights the rare presentation of HLH with acute liver failure. Although liver involvement is common, acute liver failure with HLH is very rare [7]. Presentation of a patient with acute liver injury without prior history of any liver insults including chronic alcohol use and negative hepatitis panel should prompt clinicians to look for less common diagnoses including HLH.

There are currently no guidelines for the treatment of HLH secondary to malignancy and no consensus on whether treatment should initially be aimed at malignancy or HLH. In these cases, treatment is usually based on clinical expertise [2]. We were not involved in the treatment of our patient as he was transferred to another facility for chemotherapy. In general, treatment for HLH is outlined by the HLH-94 trial. The HLH-94 protocol outlines induction therapy for eight weeks with etoposide, dexamethasone, and intrathecal methotrexate to suppress the overactive inflammatory response in the body secondary to HLH. At the end of the eight weeks of induction therapy, patients are weaned off or transitioned to continuation therapy as a bridge to hematopoietic stem cell transplantation, which is the definitive therapy [8].

HLH has a mortality rate of about 41%, and prognosis depends on the cause of HLH [2]. Prognosis is poor for HLH secondary to malignancy (especially T-cell and NK-cell malignancies) and better for HLH secondary to infections and autoimmune diseases. Prognosis is worse in patients with older age, underlying lymphoma, and thrombocytopenia, and there is a higher chance of survival in patients who receive etoposide therapy [9]. One study used ferritin levels as a measure of prognosis and found that a slower rate of decline in ferritin levels (<50%) during therapy, as opposed to 96% or greater, predicted a higher mortality rate [10]. Even though our patient had HLH secondary to malignancy, the prognosis was better because it was B-cell leukemia instead of T-cell leukemia or lymphoma. The patient was relatively young and did not have significant thrombocytopenia which could possibly portend a better prognosis.

## Conclusions

HLH is a hyperactivation syndrome with a severe uncontrolled inflammatory response that can be due to primary or secondary causes. Secondary HLH is most commonly due to infections or hemotological malignancies. HLH rarely presents secondary to B-cell malignancies. In this paper, we highlight a case of HLH complicated with fulminant hepatic failure that was found to be secondary to acute B-cell lymphocytic leukemia. This condition is fatal if not identified and treated promptly. Delay in diagnosis should not be made in patients who present with vague symptoms. HLH should be on list of differentials in patients who present with unremitting fevers despite a negative infectious workup and treatment with broad-spectrum antibiotics. Higher suspicion for HLH should be considered in patients with hepatic involvement. Further investigations should be made to find the source of HLH. This case is unique because the patient presented with liver failure secondary to HLH. Although liver injury is common, acute liver failure in HLH is very rare. HLH should be considered as a diagnosis in patients who present with acute liver failure without any prior history of liver injury or in those without an indeterminate cause. This case report can add to the literature describing the various presentations of HLH, and urging clinicians to be aware of this multisystem disease and its multifaceted presentation.
